# Management of Asymptomatic Silicone Breast Implant Rupture in Elderly Patients: A Scoping Review and Proposed Guidelines

**DOI:** 10.1093/asjof/ojag138.005

**Published:** 2026-07-24

**Authors:** N A Vernice, C J Boyd, K Hemal, C Amro, T J Sorenson, J Park, N Karp, O Cohen, M Choi

## Abstract

**Goals/Purpose:**

Breast implants are a cornerstone of aesthetic and reconstructive breast surgery, with over 3.5 million recipients in the United States. Implant rupture risk increases with device age, though modern cohesive silicone implants exhibit considerably improved durability. The U.S. Food and Drug Administration (FDA) does not recommend routine implant replacement but advises surveillance screening for silicone implant rupture beginning 5–6 years after placement and every 2–3 years thereafter. Despite these recommendations, no guidelines exist for managing asymptomatic ruptures in elderly patients, a population with distinct physiological risks and reduced life expectancy. This study aimed to evaluate existing literature and propose practical, evidence-informed recommendations for the management of asymptomatic silicone implant rupture in elderly patients, balancing surgical risk, longevity, and quality of life.

**Methods/Technique:**

A comprehensive scoping review was performed through January 2025 using MEDLINE (Ovid), EMBASE, and the Cochrane Library. Search terms included breast implant rupture, breast implant failure, elderly, geriatric, and older patient. There were no restrictions on publication date or study type to minimize selection bias. Articles were screened for the presence of published guidelines addressing management of asymptomatic breast implant rupture in elderly patients. None were identified. In the absence of existing recommendations, the authors analyzed available evidence on rupture surveillance, surgical risk in elderly cohorts, and geriatric decision frameworks. A modified Comprehensive Geriatric Assessment (CGA) tool was developed to assist plastic surgeons in evaluating surgical candidacy, incorporating frailty, comorbidities, nutritional status, metabolic fitness, and operative risk.

**Results/Complications:**

The authors acknowledge that surveillance imaging (MRI or ultrasound) can detect silent ruptures and minimize silicone migration. However, the clinical relevance of early rupture detection in elderly patients is limited. Modern cohesive gel implants rarely produce symptomatic migration, and intracapsular ruptures are typically inert. No data link silicone leakage to systemic disease or connective tissue disorders. Moreover, routine surveillance carries the risks of false positives, overdiagnosis, and unnecessary surgery. MRI, while the gold standard, is costly and may identify clinically asymptomatic ruptures. Ultrasound, though accessible, has high operator dependency and non-negligible false-positive rates. Overdiagnosis—defined as detection of a rupture that would not become symptomatic during the patient’s lifetime—can prompt anxiety and unwarranted surgery. Elderly patients also face increased perioperative risk. More specifically, studies in implant-based reconstruction show postoperative complication rates of 27.9% in patients ≥70 years compared to 14.8% in younger counterparts. Advanced age is further associated with impaired wound healing, frailty, and polypharmacy-related risk. Based on the synthesis of evidence and analogous screening principles (e.g., breast cancer surveillance discontinuation after age 75 per the United States Preventative Services Task Force), the authors propose to discontinue surveillance screening for asymptomatic silicone implants after age 75, forego routine implant exchange in asymptomatic patients >75 years, and observe asymptomatic patients age >75 years with known or suspected rupture, reserving imaging for new symptoms or physical changes. Ultimately, surgeons should engage patients in discussions that integrate their tolerance for uncertainty, surgical risk, and aesthetic priorities. Routine imaging reports of “suspected rupture” may heighten anxiety and perceived pain, sometimes precipitating unnecessary surgery. Surgeons are advised to counsel patients against reflexive explantation in the absence of symptoms.

**Conclusion:**

Routine surveillance and surgical intervention for asymptomatic silicone breast implant rupture in elderly patients may not be warranted. The risks of false positives, heightened anxiety, and perioperative complications outweigh the limited benefits of detecting clinically benign ruptures in patients with reduced life expectancy. The authors recommend discontinuing surveillance screening and routine implant exchange after age 75, reserving intervention for symptomatic cases only. A shared decision-making model—incorporating comprehensive geriatric assessment, patient comfort with uncertainty, and aesthetic goals—should guide management. These recommendations aim to optimize quality of life and avoid overtreatment in elderly patients living with breast implants.

